# *N*′-[(*E*)-2-Chloro­benzyl­idene]-2-(6-meth­oxy­naphthalen-2-yl)propano­hydrazide

**DOI:** 10.1107/S1600536814008629

**Published:** 2014-05-03

**Authors:** Joel T. Mague, Shaaban K. Mohamed, Mehmet Akkurt, Herman Potgieter, Mustafa R. Albayati

**Affiliations:** a Department of Chemistry, Tulane University, New Orleans, LA 70118, USA; b Chemistry and Environmental Division, Manchester Metropolitan University, Manchester M1 5GD, England; c Chemistry Department, Faculty of Science, Minia University, 61519 El-Minia, Egypt; d Department of Physics, Faculty of Sciences, Erciyes University, 38039 Kayseri, Turkey; e Analytical Development Division, Manchester Metropolitan University, Manchester M1 5GD, England; f Kirkuk University, College of Science, Department of Chemistry, Kirkuk, Iraq

## Abstract

In the title compound, C_21_H_19_ClN_2_O_2_, the benzene ring and the naphthalene ring system are oriented at a dihedral angle of 65.24 (10)°. In the crystal, N—H⋯O, C—H⋯N and C—H⋯O hydrogen bonds link the mol­ecules, forming chains along the *b*-axis direction. Further C—H⋯O hydrogen bonds link the chains, forming corrugated sheets lying parallel to (10-1).

## Related literature   

For the use of Naproxen [systematic name: (+)-(*S*)-2-(6-meth­oxy­naphthalen-2-yl)propanoic acid] and hydrazide-hydrazones in the treatment of disease and inflammation, see: Merlet *et al.* (2013 ▶); Almasirad *et al.* (2005 ▶, 2006 ▶). For the harmful side-effects of non-steroidal anti-inflammatory drugs (NSAIDs), see: Uzgören-Baran *et al.* (2012 ▶); Tozkoparan *et al.* (2012 ▶). For the synthesis of NSAIDS with safer pro-drug profiles and enhanced chromphore efficacy, see: Koopaei *et al.* (2013 ▶). For bond-length data, see: Allen *et al.* (1987 ▶).
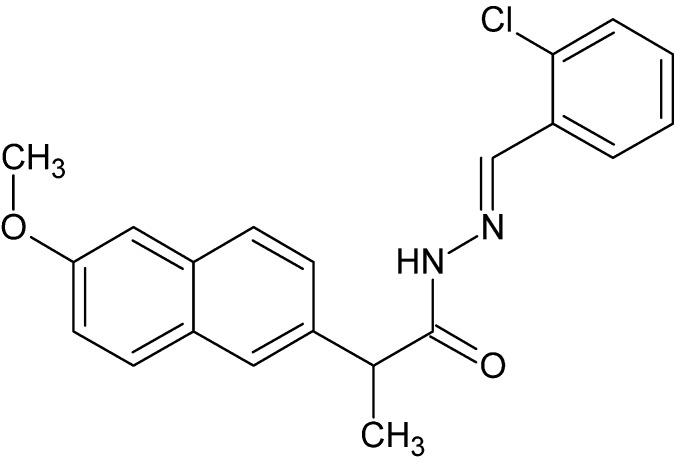


## Experimental   

### 

#### Crystal data  

C_21_H_19_ClN_2_O_2_
*M* *_r_* = 366.83
Monoclinic, 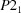

*a* = 6.5703 (2) Å
*b* = 8.6166 (2) Å
*c* = 16.3411 (4) Å
β = 98.6850 (9)°
*V* = 914.52 (4) Å^3^
*Z* = 2
Cu *K*α radiation
μ = 1.99 mm^−1^
*T* = 100 K
0.23 × 0.09 × 0.02 mm

#### Data collection  

Bruker D8 VENTURE PHOTON 100 CMOS diffractometer
Absorption correction: multi-scan (*SADABS*; Bruker, 2013 ▶) *T*_min_ = 0.87, *T*_max_ = 0.96
13492 measured reflections
3243 independent reflections
3122 reflections with *I* > 2σ(*I*)
*R*_int_ = 0.030

#### Refinement  

*R*[*F* ^2^ > 2σ(*F* ^2^)] = 0.026
*wR*(*F* ^2^) = 0.064
*S* = 1.05
3243 reflections
237 parameters
1 restraint
H-atom parameters constrained
Δρ_max_ = 0.20 e Å^−3^
Δρ_min_ = −0.12 e Å^−3^
Absolute structure: Flack parameter determined using 1358 quotients [(*I* ^+^)−(*I* ^−^)]/[(*I* ^+^)+(*I* ^−^)] (Parsons *et al.*, 2013 ▶)
Absolute structure parameter: 0.056 (5)

### 

Data collection: *APEX2* (Bruker, 2013 ▶); cell refinement: *SAINT* (Bruker, 2013 ▶); data reduction: *SAINT*; program(s) used to solve structure: *SHELXT* (Sheldrick, 2008 ▶); program(s) used to refine structure: *SHELXL* (Sheldrick, 2008 ▶); molecular graphics: *DIAMOND* (Brandenburg & Putz, 2012 ▶); software used to prepare material for publication: *SHELXTL* (Sheldrick, 2008 ▶).

## Supplementary Material

Crystal structure: contains datablock(s) global, I. DOI: 10.1107/S1600536814008629/sj5398sup1.cif

Structure factors: contains datablock(s) I. DOI: 10.1107/S1600536814008629/sj5398Isup2.hkl

Supporting information file. DOI: 10.1107/S1600536814008629/sj5398Isup3.cml

CCDC reference: 997601

Additional supporting information:  crystallographic information; 3D view; checkCIF report

## Figures and Tables

**Table 1:** Hydrogen-bond geometry (Å, °)

*D*—H⋯*A*	*D*—H	H⋯*A*	*D*⋯*A*	*D*—H⋯*A*
N1—H1⋯O2^i^	0.91	1.93	2.829 (2)	169
C12—H12⋯N2^i^	1.00	2.48	3.444 (3)	163
C15—H15⋯O2^i^	0.95	2.48	3.259 (3)	139
C19—H19⋯O1^ii^	0.95	2.57	3.486 (3)	163

Symmetry codes: (i) 
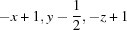
; (ii) 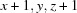.

**Fig. 1.**
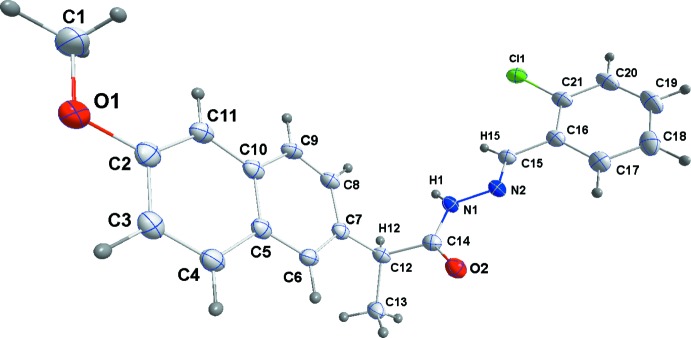
A perspective view of the title molecule with 50% probability ellipsoids.

**Fig. 2.**
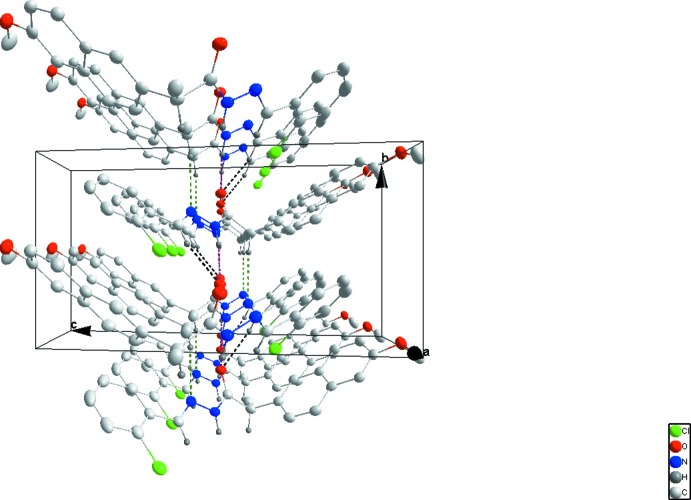
Crystal packing viewed down the a axis showing hydrogen bonds as dotted lines (N—H···O: purple, C—H···O: black, C—H···N: green).

## supplementary crystallographic information

### 1. Comment

Naproxen ((+)-(S)-2-(6-methoxynaphthalen-2-yl)propanoic acid), among many non-steroidal anti-inflammatory drugs (NSAIDs), is used mainly in the treatment of pain, rehumatiod and inflammatory diseases (Merlet *et al.*, 2013). It was reported that the presence of a carboxylic acid group in the parent drug leads to many undesirable side-effects such as gastrointestinal toxicity and ulceration (Uzgören-Baran *et al.*, 2012; Tozkoparan *et al.*, 2012). Recently, it was found that masking the carboxylic acid residue in the parent drug of NSAIDs led to safer pro-drug profiles and enhanced the chromophore efficacy (Koopaei *et al.*, 2013). On the other hand, hydrazide-hydrazone scaffold compounds have been found to possess significant anti-inflammatory effects (Almasirad *et al.*, 2005; Almasirad *et al.*, 2006). Based on these findings the title compound was designed to be a hydrazone profile incorporating the Naproxen core structure without a carboxylic acid substituent.

The naphthalene ring system of the title compound (I, Fig.-1) is essentialy planar [maximum deviation = 0.025 (2) Å for C7 and -0.022 (2) Å for C9]. The dihedral angle between the mean planes of the naphthalene and phenyl groups is 65.24 (10)°. The C1–O1–C2–C11, C6–C7–C12–C13, C7–C12–C14–O2, C7–C12–C14–N1, C12–C14–N1–N2, O2–C14–N1–N2 and C14–N1–N2–C15 torsion angles are -3.6 (3), 28.5 (3), 82.8 (2), -95.9 (2), 177.67 (18), -3.6 (3) and 177.4 (2) °, respectively. In (I), the bond lengths (Allen *et al.*, 1987) and angles are within normal ranges.

In the crystal structure, the N—H···O and C—H···N hydrogen bonds link the molecules, forming chains along the b-axis (Table 1 and Fig. 2). However, sensible C—H···O contacts are also present that link molecules into chains along *c* and extend the packing along the *c* axis.

### 2. Experimental

A mixture of 1 mmol (244 mg) Naproxen acid hydrazide [2-(6-methoxy-2-naphthyl)propanehydrazide] and 1 mmol (141 mg) 2-chlorobenzaldehyde was refluxed in 30 ml ethanol for 5hr in the presence of a few catalytic drops of glacial acetic acid. The mixture was cooled and separated, the solid filtered off, dried under vacuum and recrytallized from ethanol to furnish white crystals in a good quality suitable for X-ray diffraction. Mp 488–451 K.

### 3. Refinement

H-atoms attached to carbon were placed in calculated positions (C—H = 0.95 - 1.00 Å) while those attached to nitrogen were placed in locations derived from a difference map and their parameters adjusted to give N—H = 0.91 Å. All were included as riding contributions with isotropic displacement parameters 1.2 - 1.5 times those of the attached atoms.

### Crystal data

**Table:**

C_21_H_19_ClN_2_O_2_	*F*(000) = 384
*M**_r_* = 366.83	*D*_x_ = 1.332 Mg m^−^^3^
Monoclinic, *P*2_1_	Cu *K*α radiation, λ = 1.54178 Å
Hall symbol: P 2yb	Cell parameters from 9961 reflections
*a* = 6.5703 (2) Å	θ = 2.7–68.2°
*b* = 8.6166 (2) Å	µ = 1.99 mm^−^^1^
*c* = 16.3411 (4) Å	*T* = 100 K
β = 98.6850 (9)°	Plate, colourless
*V* = 914.52 (4) Å^3^	0.23 × 0.09 × 0.02 mm
*Z* = 2	

### Data collection

**Table:**

Bruker D8 VENTURE PHOTON 100 CMOS diffractometer	3243 independent reflections
Radiation source: INCOATEC IµS micro–focus source	3122 reflections with *I* > 2σ(*I*)
Mirror monochromator	*R*_int_ = 0.030
Detector resolution: 10.4167 pixels mm^-1^	θ_max_ = 68.2°, θ_min_ = 2.7°
ω and φ scans	*h* = −7→7
Absorption correction: multi-scan (*SADABS*; Bruker, 2013)	*k* = −9→10
*T*_min_ = 0.87, *T*_max_ = 0.96	*l* = −19→19
13492 measured reflections	

### Refinement

**Table:**

Refinement on *F*^2^	Secondary atom site location: difference Fourier map
Least-squares matrix: full	Hydrogen site location: mixed
*R*[*F*^2^ > 2σ(*F*^2^)] = 0.026	H-atom parameters constrained
*wR*(*F*^2^) = 0.064	*w* = 1/[σ^2^(*F*_o_^2^) + (0.0303*P*)^2^ + 0.1372*P*] where *P* = (*F*_o_^2^ + 2*F*_c_^2^)/3
*S* = 1.05	(Δ/σ)_max_ = 0.001
3243 reflections	Δρ_max_ = 0.20 e Å^−^^3^
237 parameters	Δρ_min_ = −0.12 e Å^−^^3^
1 restraint	Absolute structure: Flack parameter determined using 1358 quotients [(*I*^+^)-(*I*^-^)]/[(*I*^+^)+(*I*^-^)] (Parsons *et al.*, 2013)
Primary atom site location: structure-invariant direct methods	Absolute structure parameter: 0.056 (5)

### Special details

**Table:**

Geometry. Bond distances, angles *etc*. have been calculated using the rounded fractional coordinates. All su's are estimated from the variances of the (full) variance-covariance matrix. The cell e.s.d.'s are taken into account in the estimation of distances, angles and torsion angles
Refinement. Refinement on *F*^2^ for ALL reflections except those flagged by the user for potential systematic errors. Weighted *R*-factors *wR* and all goodnesses of fit *S* are based on *F*^2^, conventional *R*-factors *R* are based on *F*, with *F* set to zero for negative *F*^2^. The observed criterion of *F*^2^ > σ(*F*^2^) is used only for calculating -*R*-factor-obs *etc*. and is not relevant to the choice of reflections for refinement. *R*-factors based on *F*^2^ are statistically about twice as large as those based on *F*, and *R*-factors based on ALL data will be even larger.

### Fractional atomic coordinates and isotropic or equivalent isotropic displacement parameters (Å^2^)

**Table:**

	*x*	*y*	*z*	*U*_iso_*/*U*_eq_	
Cl1	1.26515 (8)	0.49714 (7)	0.64924 (3)	0.0316 (2)	
O1	0.4688 (2)	1.0112 (2)	−0.00565 (9)	0.0298 (5)	
O2	0.3102 (2)	0.82203 (18)	0.49852 (9)	0.0270 (5)	
N1	0.5530 (3)	0.6307 (2)	0.51581 (11)	0.0228 (6)	
N2	0.6679 (3)	0.7022 (2)	0.58251 (11)	0.0244 (6)	
C1	0.6649 (4)	0.9724 (4)	−0.02662 (17)	0.0422 (9)	
C2	0.4163 (3)	0.9427 (3)	0.06423 (14)	0.0246 (7)	
C3	0.2255 (3)	0.9925 (3)	0.08485 (12)	0.0251 (6)	
C4	0.1563 (3)	0.9358 (3)	0.15342 (14)	0.0255 (7)	
C5	0.2726 (3)	0.8254 (3)	0.20542 (13)	0.0217 (6)	
C6	0.2070 (3)	0.7663 (3)	0.27802 (14)	0.0227 (7)	
C7	0.3221 (3)	0.6596 (2)	0.32769 (14)	0.0220 (6)	
C8	0.5082 (3)	0.6063 (3)	0.30327 (14)	0.0245 (7)	
C9	0.5762 (3)	0.6609 (3)	0.23431 (14)	0.0246 (7)	
C10	0.4627 (3)	0.7741 (2)	0.18331 (14)	0.0227 (6)	
C11	0.5318 (3)	0.8356 (3)	0.11192 (14)	0.0235 (6)	
C12	0.2624 (3)	0.5983 (3)	0.40812 (13)	0.0231 (7)	
C13	0.0309 (3)	0.5967 (3)	0.41077 (14)	0.0272 (7)	
C14	0.3743 (3)	0.6955 (2)	0.47855 (14)	0.0226 (7)	
C15	0.8290 (3)	0.6268 (3)	0.61428 (14)	0.0236 (7)	
C16	0.9609 (3)	0.6871 (3)	0.68768 (14)	0.0243 (7)	
C17	0.8874 (4)	0.7958 (3)	0.73949 (14)	0.0294 (8)	
C18	1.0113 (4)	0.8504 (3)	0.80960 (15)	0.0353 (8)	
C19	1.2109 (4)	0.7965 (3)	0.82972 (16)	0.0387 (9)	
C20	1.2888 (4)	0.6892 (3)	0.77994 (16)	0.0330 (8)	
C21	1.1634 (4)	0.6351 (3)	0.70945 (15)	0.0276 (7)	
H1	0.58750	0.53150	0.50470	0.0270*	
H1A	0.77230	0.99770	0.01990	0.0630*	
H1B	0.68830	1.03170	−0.07550	0.0630*	
H1C	0.66940	0.86110	−0.03860	0.0630*	
H3	0.14500	1.06600	0.05070	0.0300*	
H4	0.02800	0.97060	0.16660	0.0310*	
H6	0.08020	0.80120	0.29270	0.0270*	
H8	0.58740	0.53030	0.33610	0.0290*	
H9	0.70180	0.62280	0.22000	0.0300*	
H11	0.65930	0.80210	0.09730	0.0280*	
H12	0.31390	0.48930	0.41570	0.0280*	
H13A	0.00370	0.53980	0.45990	0.0410*	
H13B	−0.04030	0.54570	0.36090	0.0410*	
H13C	−0.01910	0.70350	0.41320	0.0410*	
H15	0.86290	0.53170	0.59020	0.0280*	
H17	0.75010	0.83280	0.72640	0.0350*	
H18	0.95920	0.92500	0.84390	0.0420*	
H19	1.29500	0.83360	0.87820	0.0460*	
H20	1.42620	0.65270	0.79360	0.0400*	

### Atomic displacement parameters (Å^2^)

**Table:**

	*U*^11^	*U*^22^	*U*^33^	*U*^12^	*U*^13^	*U*^23^
Cl1	0.0219 (3)	0.0340 (3)	0.0383 (3)	0.0029 (2)	0.0027 (2)	0.0080 (3)
O1	0.0250 (8)	0.0373 (9)	0.0268 (8)	0.0025 (8)	0.0030 (6)	0.0061 (8)
O2	0.0245 (8)	0.0235 (8)	0.0314 (9)	0.0027 (7)	−0.0005 (6)	0.0002 (7)
N1	0.0203 (10)	0.0203 (9)	0.0262 (10)	0.0012 (7)	−0.0014 (7)	−0.0004 (7)
N2	0.0211 (9)	0.0251 (10)	0.0256 (10)	−0.0019 (7)	−0.0007 (8)	0.0014 (8)
C1	0.0297 (13)	0.0577 (19)	0.0408 (14)	0.0070 (12)	0.0104 (11)	0.0197 (13)
C2	0.0245 (12)	0.0266 (12)	0.0214 (11)	−0.0034 (8)	−0.0003 (9)	−0.0032 (8)
C3	0.0248 (11)	0.0244 (10)	0.0242 (10)	0.0035 (10)	−0.0023 (8)	0.0000 (10)
C4	0.0208 (11)	0.0283 (11)	0.0260 (12)	0.0053 (9)	−0.0012 (9)	−0.0028 (9)
C5	0.0204 (11)	0.0210 (11)	0.0224 (11)	0.0010 (9)	−0.0011 (8)	−0.0044 (9)
C6	0.0168 (11)	0.0247 (11)	0.0258 (12)	0.0018 (8)	0.0009 (9)	−0.0050 (9)
C7	0.0193 (11)	0.0215 (11)	0.0242 (11)	−0.0021 (8)	−0.0003 (9)	−0.0037 (8)
C8	0.0199 (11)	0.0211 (11)	0.0310 (12)	0.0018 (8)	−0.0005 (9)	0.0013 (9)
C9	0.0168 (11)	0.0235 (11)	0.0331 (13)	0.0031 (8)	0.0028 (9)	−0.0031 (9)
C10	0.0198 (11)	0.0213 (11)	0.0258 (11)	−0.0007 (8)	−0.0005 (9)	−0.0065 (8)
C11	0.0195 (11)	0.0240 (11)	0.0267 (11)	0.0008 (9)	0.0023 (9)	−0.0038 (9)
C12	0.0190 (11)	0.0212 (11)	0.0279 (12)	0.0016 (8)	−0.0001 (9)	0.0021 (9)
C13	0.0211 (12)	0.0328 (13)	0.0272 (12)	−0.0011 (9)	0.0016 (10)	0.0029 (10)
C14	0.0194 (11)	0.0225 (11)	0.0261 (12)	0.0002 (8)	0.0039 (9)	0.0026 (9)
C15	0.0215 (11)	0.0234 (11)	0.0258 (12)	−0.0004 (9)	0.0033 (9)	0.0032 (9)
C16	0.0252 (12)	0.0234 (12)	0.0237 (11)	−0.0035 (8)	0.0016 (9)	0.0054 (8)
C17	0.0321 (13)	0.0267 (13)	0.0285 (13)	−0.0022 (9)	0.0019 (10)	0.0045 (9)
C18	0.0487 (16)	0.0289 (14)	0.0279 (13)	−0.0086 (11)	0.0045 (11)	−0.0003 (10)
C19	0.0438 (16)	0.0399 (16)	0.0281 (13)	−0.0181 (12)	−0.0080 (11)	0.0046 (11)
C20	0.0269 (12)	0.0356 (14)	0.0328 (14)	−0.0107 (10)	−0.0071 (10)	0.0107 (10)
C21	0.0250 (12)	0.0283 (13)	0.0285 (12)	−0.0051 (9)	0.0006 (10)	0.0092 (9)

### Geometric parameters (Å, º)

**Table:**

Cl1—C21	1.740 (3)	C16—C17	1.397 (3)
O1—C1	1.422 (3)	C17—C18	1.384 (3)
O1—C2	1.375 (3)	C18—C19	1.383 (4)
O2—C14	1.231 (2)	C19—C20	1.380 (4)
N1—N2	1.374 (3)	C20—C21	1.392 (4)
N1—C14	1.358 (3)	C1—H1A	0.9800
N2—C15	1.283 (3)	C1—H1B	0.9800
N1—H1	0.9100	C1—H1C	0.9800
C2—C11	1.362 (3)	C3—H3	0.9500
C2—C3	1.413 (3)	C4—H4	0.9500
C3—C4	1.362 (3)	C6—H6	0.9500
C4—C5	1.419 (3)	C8—H8	0.9500
C5—C6	1.417 (3)	C9—H9	0.9500
C5—C10	1.422 (3)	C11—H11	0.9500
C6—C7	1.375 (3)	C12—H12	1.0000
C7—C12	1.522 (3)	C13—H13A	0.9800
C7—C8	1.419 (3)	C13—H13B	0.9800
C8—C9	1.358 (3)	C13—H13C	0.9800
C9—C10	1.419 (3)	C15—H15	0.9500
C10—C11	1.417 (3)	C17—H17	0.9500
C12—C13	1.528 (3)	C18—H18	0.9500
C12—C14	1.520 (3)	C19—H19	0.9500
C15—C16	1.465 (3)	C20—H20	0.9500
C16—C21	1.398 (3)		
			
C1—O1—C2	116.70 (19)	Cl1—C21—C20	117.8 (2)
N2—N1—C14	120.35 (17)	O1—C1—H1A	109.00
N1—N2—C15	114.50 (18)	O1—C1—H1B	109.00
N2—N1—H1	117.00	O1—C1—H1C	110.00
C14—N1—H1	121.00	H1A—C1—H1B	109.00
O1—C2—C3	114.2 (2)	H1A—C1—H1C	109.00
O1—C2—C11	125.31 (19)	H1B—C1—H1C	110.00
C3—C2—C11	120.5 (2)	C2—C3—H3	120.00
C2—C3—C4	120.4 (2)	C4—C3—H3	120.00
C3—C4—C5	121.10 (19)	C3—C4—H4	119.00
C6—C5—C10	119.4 (2)	C5—C4—H4	119.00
C4—C5—C6	122.54 (19)	C5—C6—H6	119.00
C4—C5—C10	118.05 (19)	C7—C6—H6	119.00
C5—C6—C7	121.66 (19)	C7—C8—H8	119.00
C6—C7—C8	118.1 (2)	C9—C8—H8	119.00
C6—C7—C12	123.46 (18)	C8—C9—H9	119.00
C8—C7—C12	118.43 (18)	C10—C9—H9	119.00
C7—C8—C9	121.8 (2)	C2—C11—H11	120.00
C8—C9—C10	121.1 (2)	C10—C11—H11	120.00
C5—C10—C11	119.72 (19)	C7—C12—H12	108.00
C9—C10—C11	122.36 (19)	C13—C12—H12	108.00
C5—C10—C9	117.92 (19)	C14—C12—H12	108.00
C2—C11—C10	120.28 (19)	C12—C13—H13A	109.00
C7—C12—C14	107.72 (18)	C12—C13—H13B	109.00
C7—C12—C13	114.52 (18)	C12—C13—H13C	110.00
C13—C12—C14	110.68 (18)	H13A—C13—H13B	109.00
N1—C14—C12	113.54 (17)	H13A—C13—H13C	109.00
O2—C14—C12	122.90 (19)	H13B—C13—H13C	110.00
O2—C14—N1	123.55 (19)	N2—C15—H15	120.00
N2—C15—C16	120.1 (2)	C16—C15—H15	120.00
C17—C16—C21	117.6 (2)	C16—C17—H17	119.00
C15—C16—C17	121.2 (2)	C18—C17—H17	119.00
C15—C16—C21	121.2 (2)	C17—C18—H18	120.00
C16—C17—C18	121.1 (2)	C19—C18—H18	120.00
C17—C18—C19	120.1 (2)	C18—C19—H19	120.00
C18—C19—C20	120.5 (2)	C20—C19—H19	120.00
C19—C20—C21	119.1 (2)	C19—C20—H20	120.00
C16—C21—C20	121.7 (2)	C21—C20—H20	120.00
Cl1—C21—C16	120.48 (19)		
			
C1—O1—C2—C3	176.4 (2)	C8—C7—C12—C13	−152.7 (2)
C1—O1—C2—C11	−3.6 (3)	C8—C7—C12—C14	83.7 (2)
C14—N1—N2—C15	177.4 (2)	C7—C8—C9—C10	−0.2 (4)
N2—N1—C14—O2	3.6 (3)	C8—C9—C10—C5	−1.7 (3)
N2—N1—C14—C12	−177.67 (18)	C8—C9—C10—C11	178.7 (2)
N1—N2—C15—C16	−177.67 (19)	C5—C10—C11—C2	−0.4 (3)
O1—C2—C3—C4	−178.9 (2)	C9—C10—C11—C2	179.2 (2)
C11—C2—C3—C4	1.0 (4)	C7—C12—C14—O2	82.8 (2)
O1—C2—C11—C10	179.3 (2)	C7—C12—C14—N1	−95.9 (2)
C3—C2—C11—C10	−0.7 (4)	C13—C12—C14—O2	−43.1 (3)
C2—C3—C4—C5	−0.3 (4)	C13—C12—C14—N1	138.2 (2)
C3—C4—C5—C6	178.8 (2)	N2—C15—C16—C17	20.3 (3)
C3—C4—C5—C10	−0.8 (3)	N2—C15—C16—C21	−161.3 (2)
C4—C5—C6—C7	−179.9 (2)	C15—C16—C17—C18	178.8 (2)
C10—C5—C6—C7	−0.4 (3)	C21—C16—C17—C18	0.3 (4)
C4—C5—C10—C9	−178.5 (2)	C15—C16—C21—Cl1	0.3 (3)
C4—C5—C10—C11	1.1 (3)	C15—C16—C21—C20	−178.7 (2)
C6—C5—C10—C9	2.0 (3)	C17—C16—C21—Cl1	178.83 (19)
C6—C5—C10—C11	−178.4 (2)	C17—C16—C21—C20	−0.2 (4)
C5—C6—C7—C8	−1.5 (3)	C16—C17—C18—C19	−0.5 (4)
C5—C6—C7—C12	177.2 (2)	C17—C18—C19—C20	0.6 (4)
C6—C7—C8—C9	1.8 (3)	C18—C19—C20—C21	−0.4 (4)
C12—C7—C8—C9	−177.0 (2)	C19—C20—C21—Cl1	−178.8 (2)
C6—C7—C12—C13	28.5 (3)	C19—C20—C21—C16	0.2 (4)
C6—C7—C12—C14	−95.1 (2)		

### Hydrogen-bond geometry (Å, º)

**Table:**

*D*—H···*A*	*D*—H	H···*A*	*D*···*A*	*D*—H···*A*
N1—H1···O2^i^	0.91	1.93	2.829 (2)	169
C12—H12···N2^i^	1.00	2.48	3.444 (3)	163
C15—H15···O2^i^	0.95	2.48	3.259 (3)	139
C19—H19···O1^ii^	0.95	2.57	3.486 (3)	163

Symmetry codes: (i) −*x*+1, *y*−1/2, −*z*+1; (ii) *x*+1, *y*, *z*+1.
